# A Bibliometric Analysis of Child Language During 1900–2021

**DOI:** 10.3389/fpsyg.2022.862042

**Published:** 2022-06-08

**Authors:** Xingrong Guo

**Affiliations:** College of Foreign Languages, Shanghai Maritime University, Shanghai, China

**Keywords:** child language, bibliometric analysis, VOSviewer, CiteSpace, visualization

## Abstract

This study purposed to provide a bibliometric overview of child language (CL) research from 1900 to 2021 and identify major trends in CL. A total of 48,453 research articles related to the CL were identified from the Web of Science. Co-authorship, co-word, and co-citation analysis was conducted by using VOSviewer and CiteSpace. The following was analyzed: annual distribution of related papers; related disciplines; mainstream journals; geographical and institutional distribution; hot topics; keyword burst detection; and co-citation analysis of journals, authors, and references. Results showed that, under the impact of new empirical methods and new theories, the field of CL is undergoing great changes. Research hotspot and the research trends mainly concentrated on autism spectrum disorder, school readiness, oral language, reading comprehension, exposure, bilingualism, vocabulary, input, skills, kindergarten, cochlear implants, and intervention. More and more pieces of research focus on the individual difference in CL development and the importance of intervention in language education by typically developing children and some children with disabilities or language disorders. Besides, child second language acquisition also attracted a lot of attention. This bibliometric analysis is of great reference significance for researchers to understand the progress and discipline development trend in this field.

## Introduction

Language learning and development in early childhood is the most critical period of one’s life. The excellent way of child language (CL) acquisition has long attracted the attention of researchers. As early as the 18th century, German Philosopher Dietrich Tiedemann observed and described the psychological and linguistic development process of his child. The early research on children’s language acquisition mainly adopted the method of diary recording (e.g., Donahue, 1993). Since the 1960s, foreign psychologists and linguists have adopted empirical research methods and conducted many detailed studies on children’s language acquisition. In the past 30 years, some nativists represented by Norm Chomsky have focused on the study of principle and parameter theory. With the attention paid to the study of children’s language acquisition, many theories have been put forward. The change of a theoretical perspective has brought new questions and new methods. For example, statistical learning methods were investigated in infants’ ability to detect patterns in language input. Progress has also been achieved in understanding the relationship between cognitive and language development (e.g., Bowerman and Levinson, 2001). Interdisciplinary experimental research provides a lot of data support for theoretical construction. The increasing number of languages in cross-language research provides valuable information on the impact of language-specific factors and leads to a discussion on language universals.

The development and the progress of language acquisition research benefit from new technologies and methods, such as online testing of children’s language knowledge development (Sekerina et al., 2008). In addition, there were also studies using eye-tracking technology to tap children’s ability to process language structure online (Armstrong et al., 2016). Besides, many studies have used neurophysiological methods to detect the brain’s response to language-related stimuli (Kaga et al., 2010). Since the mid-1980s, Macwhiney and Snow have built the world’s largest children’s corpus, namely, the child language data exchange system (CHILDES). The CHILDES provides a powerful tool for children’s language research so that children’s corpus can be fully observed, described, and interpreted (MacWhinney, 2000). In the past decades, great advance has been made in the CL research.

In recent years, children’s language acquisition has gradually developed into the across-disciplinary research field, covering sociology, pedagogy, psychology, physiology, etc. Over the preceding decades, psychologists, experts, linguists, clinicians, and teachers have been researching CL trying to explain it using differing theoretical and experimental approaches. Researchers focus on various issues, such as the development of children’s language, the acquisition of children’s language ability, and the constraints of children’s language. Moreover, other issues also attracted many attentions, such as the differences between second language learning (adulthood) and first language acquisition; and whether the specific stage of language development is a common phenomenon across languages (Lust, 2006). There is abundant research that contributed to the CL acquisition in various fields. It will be hard to enumerate and summarize them one by one. In order to analyze the characteristics and development trend of children’s language acquisition research in more detail, this paper will systematically sort out and discuss the research results of children’s language acquisition by using the method of bibliometric analysis.

How children acquire language and how they process language have attracted extensive attention from governments, international organizations, and research institutions all over the world. After decades of development, research results of CL are numerous and still growing rapidly. Research methods are diverse, including cross-sectional (e.g., Dezani et al., 2010) and longitudinal studies (e.g., Trouton et al., 2002), theoretical (e.g., Ambridge and Lieven, 2011) and empirical research (Warden, 2011), and macrocosm and microcosm studies. In recent years, theoretical research and empirical research have become more popular, including longitudinal research, case study, and experimental research.

A traditional literature review will not clearly reveal the numerous networks, structures, interactions, intersections, evolution, or derivations of the various schools of thought on CL. These relationships birth new knowledge, which may not provide a hot spot analysis or indicate what frontiers to explore. The international literature develops fast and its dynamism challenges literature mining research. Bibliometric analysis can make up for the limitations of a traditional literature review and give a macroscopic overview of many pieces of academic literature (Guo X. et al., 2021). The bibliometric method mainly analyzes the bibliographic and content information in a certain field through quantitative analysis and the statistics method (Jia et al., 2014). It has been used to analyze a large body of peer-reviewed publications to evaluate the quality and the trend of publications in a specified period (Struck et al., 2021). Bibliometric analysis was usually used to evaluate the distribution patterns or contribution of authors, journals, institutions, or countries, and their cooperation relationship among them (Van Nunen et al., 2018). The most important feature of bibliometrics is that its output must be “quantity” (Guo X. R. et al., 2021). Through scientific identification, comprehensive statistics, and systematic literature collation, researchers can quickly interpret a large number of documents and extract key information (Guo et al., 2019).

Excavating the research focus developments, current hotspots, and important research directions from big data is the challenge. A macroscopic overview of CL was conducted using bibliometric analysis to achieve this goal. The following aspects were analyzed: annual distribution of related papers; the related disciplines; journal productivity; geographical and institutional distribution; hot topics; references with the strongest citation bursts; recent CL publications; and collaboration networks consisting of authors, organizations, and countries. This study tries to address the following questions:

1) What are the most influential journals and discipline distribution in the CL research field?
2) Which documents, authors, institutions, and regions are most influential in this field?
3) What are the most frequently investigated CL themes and topics?
4) What are the changes and trends in this field?

## Materials and Methods

### Data Source and Processing

**FIGURE 1:**
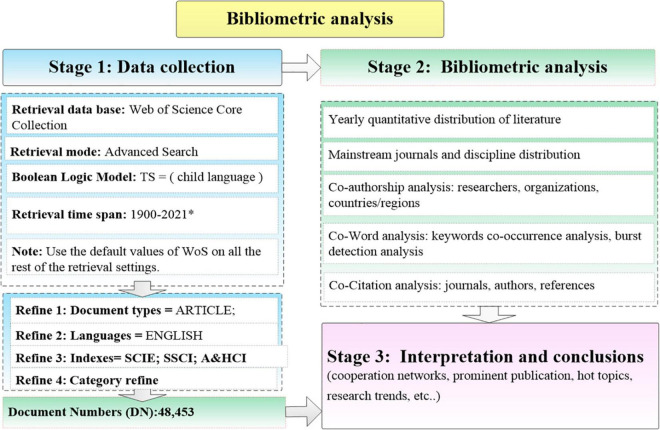
The procedure of the bibliometric analysis on CL. *The database was obtained on 10 April 2021.

The publications indexed by the Social Sciences Citation Index (SSCI, 1900–2021), the Citation Index Expanded (SCI-EXPANDED, 1900–2021), and the Arts and Humanities Citation Index (A&HCI, 1975–2021) were retrieved in the Web of Science (WoS) core collection database of Thomson Reuter on 10 April 2021. WoS is a globally recognized database, reflecting the level of scientific research. It has consistently been recognized as the world’s most authoritative scientific literature index tool, which can provide the most important research results in the field of science. SSCI, SCI, and A&HCI databases were identified as the most authoritative scientific and technological literature retrieval tools in the world, containing more than thousands of international and influential academic journals. The procedure of the bibliometric analysis is described in Figure 1.

#### Stage 1

The field tag “child language” was searched in the topic field using the advanced search command. The formula used for retrieval was: TS = child language AND LANGUAGE: English AND DOCUMENT TYPES: Article. WoS Citation Indexes: SCI-EXPANDED, SSCI, and A&HCI. There were 50,686 articles collected at first. Document types and languages were confined to articles in English. Peer-reviewed articles were chosen for bibliometric analysis because they were recognized as “have better academic standing” (Xue et al., 2020). A time span being searched was “all year” (1900 to 10 April 2021). Then, the articles were refined by categories (Supplementary Table 1). This study selected 57 categories in WoS. The categories that were more relevant to children’s language acquisition were chosen, such as linguistics, rehabilitation, developmental psychology, audiology speech language pathology, experimental psychology, etc. The categories that were less relevant to CLA were excluded, such as obstetrics gynecology, nutrition dietetics, environmental sciences, immunology, economics, etc.

There were 48,707 articles obtained by using WoS categories refine function, such as “linguistics” (11,808 articles constituting 24.24% of the total), “rehabilitation” (9,532, 19.57%), “psychology developmental” (9,108, 18.70%), “audiology speech language pathology” (6,269, 12.87%), “psychology experimental” (5,993, 12.30%), and others. Duplicate papers have been deleted by using the “remove duplicates (WoS)” function. There remained 48,707 documents. Then, irrelevant literature was excluded. Finally, there were 48,454 data used for analysis in CiteSpace and VOSviewer.

#### Stage 2

Full records and cited references of the publications were saved as plain text. The valid data obtained in Stage 1 were used for bibliometric analysis in Stage 2. The analyses were conducted: yearly quantitative distribution of the literature; mainstream journals and discipline distribution; co-authorship analysis among researchers, organizations, and countries/regions; co-word analysis: keywords co-occurrence analysis and burst detection analysis; and co-citation analysis among journals, authors, and references.

#### Stage 3

The development of CL research was overviewed. Co-operation networks, outstanding contributions, clusters, research trends, hot topics, and future directions were analyzed and reviewed.

### Analytical Methods and Procedure

As the number of documents increased, the subjectivity, incompleteness, time constraints, and other weaknesses of the traditional review become more manifested. A more scientific bibliometric method is needed to fully understand and evaluate the literature in a research field. Bibliometric analysis, which includes quantitative evaluation of scientific work, was used in this study. Various knowledge maps showed the development of scientific knowledge. Large-scale bibliometric maps display in an easy-to-interpret way (Van Eck and Waltman, 2010). Bibliometric analyses were conducted in three ways: (1) co-authorship; (2) co-word; and (3) co-citation.

VOSviewer (1.6.15) (Van Eck and Waltman, 2010) was also used to conduct co-authorship, co-occurrence, and co-citation analysis, and CiteSpace (5. 6. R5) developed by Chen (2006), which was used to generate knowledge maps, new trends, burst detection, and cluster analysis.

#### Procedure for Mainstream Journals and Co-authorship Analysis

In order to explore the relationship and cluster of the mainstream journals, the “minimum number of documents of a source” was set at 40 in VOSviewer. Precisely, 222 journals out of 2,999 sources met the thresholds. It is unusual to display thousands of nodes in a network map because too many nodes are difficult to interpret (Zhang, 2020). Usually, it is necessary to select a cutoff point to determine the number of nodes displayed in the map (McCain, 1990). For example, to better display the nodes in the map, Zhang (2020) set a cutoff point of 115 to display the top 50 most-cited authors. In order to optimize the effect of graphic display more clearly, no more than 300 nodes will be displayed in each visualization map. For example, 210 most productive authors, 167 organizations, 78 countries, 139 most popular topics, 281 most frequently cited journals, 120 authors, and 175 references were displayed in the bibliometric visualization maps.

Co-authorship analysis was utilized to explore the cooperation relationship among key researchers, organizations, countries, and regions. The settings for co-authorship analysis of VOS viewer were as follows: type of analysis: co-authorship; unit of analysis: authors; counting method: full counting; maximum number of authors per document: 25; minimum number of documents of an author: 25. Of the 103,946 authors, 210 authors met the thresholds and were used in the final network. The authors with the greatest total link strength were selected. The largest set of connecting items includes 166 items. If an author used different names in publications, it could not be merged, unless a unique digital identity strategy such as ORCID was used (Scientometrics and Hospital, 2007).

Settings for analyzing organizations in VOSviewer were as follows: analysis: co-authorship + organizations; counting method: full counting; maximum number of organizations per document: 25. Minimum number of documents of an organization: 110. Of the 16,003 organizations, 167 meet the thresholds.

The settings for analyzing countries/regions were as follows: analysis: co-authorship + countries; counting method: full counting; minimum number of documents of a country or region: 20. Of the 164 countries and regions, 78 met the thresholds.

#### Procedure for Co-word Analysis

Co-word analysis was used to detect important topics in the CL field. The settings for keywords co-occurrence network in VOSviewer were as follows: the initial step was to extract “all keywords” from titles and abstracts. A fraction counting method was used to detect 66,858 keywords. The threshold “minimum number of occurrences of a keyword” was set to 400 to create a readable map. Of the 66,858 keywords, 139 items met the threshold.

For analyzing the word burst detection, CiteSpace parameter settings were as follows: years per slice: 10; node types: keyword; term type: noun phrases + burst terms; top *N*: 50; top *N*%: 10%. For analyzing the word burst detection between 2011 and 2021, the parameter settings were as follows: years per slice: 1; node types: keywords; term type: noun phrases + burst terms; top *N* per slice: 50; and top *N*%: 10%. Approximately, 29,324 records were collected.

#### Procedure for Co-citation Analysis

Co-citation analysis was employed to discover the most cited journals, authors, and publications (Chen, 2006). The “minimum number of citations of a source” was set to 1,000 for the journal co-citation network in VOSviewer. Of the 227,965 journals, 281 met the threshold.

The “minimum number of citations of a source” was set to 1,000 for the author citation analysis. Of the 334,713 authors, 120 meet the threshold. The settings for reference co-citation analysis were as follows: types of analysis: co-citation; unit of analysis: cited reference; counting method: full counting; threshold: minimum number of citations of a cited reference: 250. Of the 869,771 cited references, 175 met the threshold.

The present study analyzed the relevant literature from the macro to the micro, from the intuitive to the complex, from the whole to the part, and from the general to the specific. Specifically, the analysis includes the literature quantitative distribution analysis; mainstream journals and discipline distribution analysis; researchers, organizations, and countries/regions analysis; hot topics analysis; and journal, author, and reference co-citation analysis.

In the similarity visualization map, the distance between two items reflects the similarity or correlation between items. Clustering methods were used to divide topics into different clusters, each with unique colors (Van Eck et al., 2010; Waltman et al., 2010). Generally, the size of the circle and the font of the label in the visualization diagram are directly proportional to the number of occurrences, and the color represents clustering (Nunen et al., 2017). The correlation and similarity between the two circles can be derived from the distance between them (Rizzi et al., 2014; Khalil and Crawford, 2015). The map can rotate and flip freely (Khalil and Crawford, 2015). In this study, journals related to children’s language were utilized to form an overlay.

## Results

### Yearly Literature Quantitative Distribution

**FIGURE 2:**
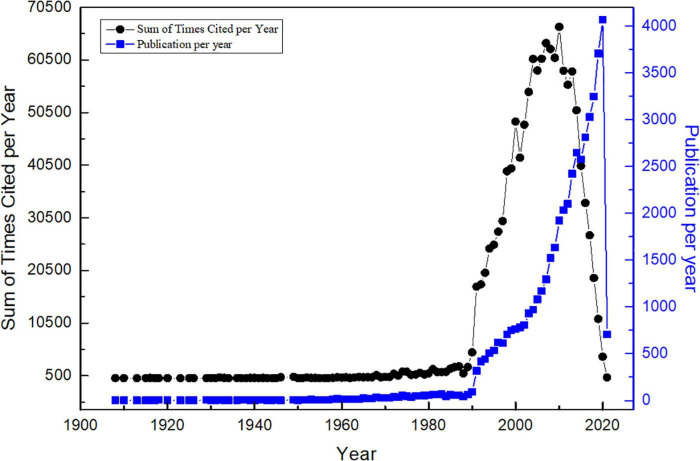
Annual distribution and citations of publications related to CL based on WoS during 1900–2021 (by 10 April 2021).

Related publications quantity distribution is a key indicator, which reveals the historical development tendency of a scientific discipline or a research field, and it will help to detect the publication trends (Guo X. R. et al., 2021). Annual distribution and citations of publications related to CL are shown in Figure 2.

Figure 2 indicates the quantity of publications from 1900 to 2021. Publication tendencies are shown in blue, and the citation trends are in black. Publications about CL were increasing as time goes by. The maximum time span that can be chosen in the WoS database is 1900–2021 in the author’s institution library. According to the advanced search in the WoS database, the term CL firstly appeared in 1908. The number of published articles was less than 100 per year before 1977 and increased slowly. From 1991 to 2002, the number of papers began to increase gradually, and, after 2003, the number of papers increased faster every year. Until now, children’s language acquisition is an issue widely concerned by all walks of life.

As shown in Figure 2, citation data shows an obvious upward trend over time. In total, the 48,453 articles were cited for 1,259,067 times until 10 April 2021, and 26 times for an average article. There were 5,126 self-citations in all. With the continuous enrichment of cross-disciplinary theories and experimental methods, the number of scientific papers on CL research will increase at a high speed.

### Mainstream Journals and Discipline Distribution

**FIGURE 3:**
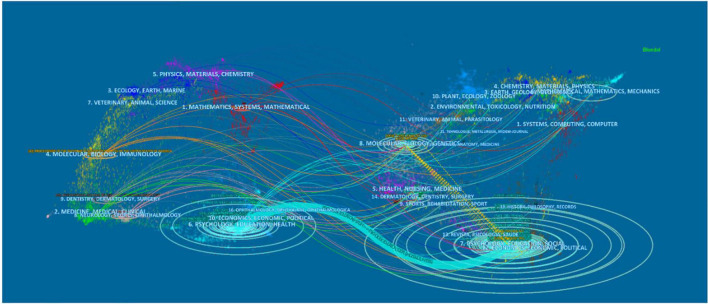
A dual-map overlay of CL literature.

A dual-map overlay map shows the discipline co-occurrence network. A dual-map overlay simultaneously displays both the citing overlay and the cited overlay (Chen and Leydesdorff, 2013). There were more than 10,000 journals on each side of the dual-map base, and they were indicated by circles. These journals belong to various disciplines in various colors and different spots of both the source field and the reference field. All the journals on the base map were clustered by the Blondel algorithm. Major clusters were labeled by terms chosen from the titles of the journals in the corresponding clusters. Taking the scientific map as the analytical base map, the periodical information of the analyzed data can be superimposed, which is helpful to understand the knowledge source of the data and the interaction of knowledge (Chen and Leydesdorff, 2013). A CL dual-map overlay of literature was conducted using CiteSpace to display the discipline co-occurrence network (Figure 3). It can display the CL literature in the context of a global scientific map.

The map on the left is the citing map, and the cited map is on the right (Figure 3). The former is the field application of the science map. The latter may be considered as a foundational research science map. Wavy curves depict citation links. The color of the links is consistent with the color of their source clusters. Connecting lines depict co-citation links across cross-disciplinary boundaries.

The dual-map overlay reveals that CL articles were published in almost all major disciplines. The links between the citing and cited matrices of journals were almost everywhere in the overlay map. For instance, the discipline “PSYCHOLOGY, EDUCATION HEALTH” is in lake-blue, and this discipline was the sixth in the source field and the seventh in the reference field. The layer containing the 48,707 bibliographic records on CL was added. Then, the source field and the reference field were linked in various colors. The links are almost everywhere in the source area, which shows that the influence of CL is widespread. Relatively, more citing journals originated from “6. PSYCHOLOGY, EDUCATION HEALTH” in the left finally link to the “7. PSYCHOLOGY, EDUCATION SOCIAL” in the cited area in the right (*z* = 1,695,203, *f* = 71,931). This indicates that “PSYCHOLOGY and EDUCATION HEALTH” are both major areas and provide a solid foundation for CL research. The rest disciplines in the citing area link to the other disciplines, such as “5. HEALTH, NURSING, MEDICINE,” “6. MATHEMATICAL, MATHEMATICS, MECHANICS,” “8. MOLECULAR, BIOLOGY GENETICS,” “14. DERMATOLOGY, DENTISTRY, SURGERY.” The dotted line represents a lot of cross-referencing between the two disciplines.

The yellow-dotted line indicates that there is a lot of cross-referencing between “7. PSYCHOLOGY, EDUCATION SOCIAL” and “8. MOLECULAR, BIOLOGY GENETICS.” Psychology is the main force of CL research. Researchers mainly use psychological methods to study children’s language in order to solve the problem of children’s psychological development. Over the years, most especially the last 20 years, CL has developed into a highly integrated interdisciplinary field. Scientists have studied this aspect of CL development in depth, combining it with a variety of disciplines, including medicine, physiology, sociology, child psychology, and pedagogy. Mainstream categories were “Linguistic, Psychology Experimental, Psychology Developmental, Rehabilitation, Language Linguistics.”

**FIGURE 4:**
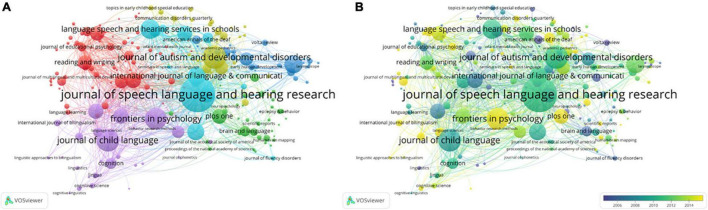
The visualization map of prominent journals. **(A)** Network visualization; **(B)** an overlay visualization map.

Academic journals are one of the important carriers of academic achievements in the scientific field. The distribution of key journals in an academic discipline can be explored by analyzing the source journal. According to citation sources results, there are 48,707 articles in 2,999 journals in total. Top 10 journals with numerous articles about CL appear in Supplementary Table 2. It showed that citations and the total publications (TP) values were important factors influencing the impact factor (IF). The visualization map of mainstream journals of CL was created by VOSviewer in Figure 4.

As shown in Figure 4, nodes represent the journals. Node size indicates the quantity of relevant documents published in a journal. Different categories are classified in different colors. Link strength between nodes represents the frequency of a citation between articles in the two journals. Mainstream journals were labeled in a larger font. As the total citations of an article of the journal increases so does its IF. The top three journals that publish most about CL are *Journal of Speech Language and Hearing Research, Child Developm*ent, and *Journal of Autism and Developmental Disorders*, with documents numbering 1,381, 499, and 830, respectively. Among the JCR categories, prominent research fields are “*Psychology, Experimental,” “Educational,”* “*Psychology, Developmental*,” and “*Linguistic*,” among the top 10 prolific journals.

### Co-authorship Analysis

Co-authors means that the researchers authored the same publication. Co-authorship network is composed of scientists with close social relations whose interaction represents collaboration or cooperation (Hurtado-Marín et al., 2021). Co-authorship network was conducted by VOSviewer to explore the co-operation relationship among the authors, organizations, and countries.

The information provided in the research records on related topics contains the relevant details of authors, which help to identify the prolific authors, institutions or colleges, and countries/regions. As a result, these data can be extended to assess co-author networks, national or regional networks, and institutional networks (Olawumi and Chan, 2018).

#### Researchers

**FIGURE 5:**
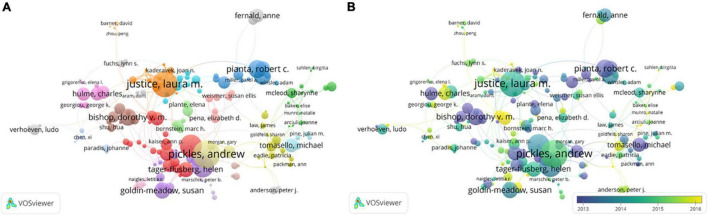
Authors’ cooperation network in the CL research field. **(A)** The co-authorship network for all items. **(B)** The co-authorship network for the largest set of items.

VOSviewer was used to explore how researchers collaborate in the CL research field. For the 103,946 authors, 24.53% have published 2 articles (*n* = 25,504); 12.11% have 3 (*n* = 12,592); 7.52% have 4 (*n* = 7,812); 5.15% have 5 (*n* = 5,355); 3.77% have 6 (*n* = 3,921); 2.9% have 7 (*n* = 3,015); 2.31% have 8 publications (*n* = 2,397); 1.86% have 9 publications (*n* = 1,934); and 1.56% have 10 publications (*n* = 1,625). Three authors have 81 publications, respectively. They are the most prolific authors in the CL field. Author cooperation networks in the CL research field appear in Figure 5.

As shown in Figure 5, a co-author network was generated with various nodes and links in different colors. The nodes of the circle represent each author, and the links connecting authors represent cooperation between them (Zhao, 2017). The node size corresponds to the number of publications per author. The thickness of the links represents the cooperative relationship between the two authors. Different colors represent different author collaboration clusters. Figure 5A shows the co-authorship network for all items. It consists of 166 items, 23 clusters, and 351 links. The total link strength is 2,048.

The largest set of items gathers in the center of the network. Figure 5B shows the co-authorship network for the largest set of items. Analysis of the main contributor’s collaboration helps to assess the current status of research. They were ranked in descending order according to publications, Bishop, D. V. M. (*n* = 60), Leonard, L. B. (*n* = 59), Justice, L. M. (*n* = 46), Conti-Ramsden, G. (*n* = 30), and Goldin-Meadow, S. (*n* = 25). The authors who were cited most were Pickles, A. (*n* = 5,186), Justice, L. M. (*n* = 4,838), Charman, T. (*n* = 4,117), Lord, C. (*n* = 4,079), and Pianta, R. C. (*n* = 3,189). Through content analysis, it is found that most of the authors with high number of articles or high citation focus on investigating the language development of children with language disorders. The 10 authors who had the most publications and strongest co-authorship are listed in Supplementary Table 3.

Three contributors with the most publications were analyzed in detail as follows: Justice, L. M. has published most articles and cited most about CL. Justice et al. (2008) were cited the most. The paper explored the quality of language and literacy instruction in preschool classrooms. Bishop, D. V. M. was interested in exploring the nature and causes of language disorders in children, and the most cited literature is Bishop and Adams (2010). The work investigated the relationship between language disorders, phonological disorders, and reading retardation. Most of Leonard L. B.’s publications are about the language development of children with language disorders, mainly on grammatical, lexical, and phonological factors. The most cited article is Leonard et al. (2007). Leonard et al. (2007) explored 14-year-old children with language impairment and their typically developing peers’ performance in both processing speed and working memory tasks. Leonard L. B.’s experiments used the paradigm of looking-while-listening (eye gaze), electrophysiological techniques, syntactic priming tasks, and more traditional comprehension and production tasks to study the nature of specific language barriers in several languages.

As the first author, the top five authors with the most papers are as follows; Justice, L. M. (*n* = 64), Bishop, D. V. M. (*n* = 60), Lawrence, L. (*n* = 59), Conti-Ramsden, G. (*n* = 30), and Goldin-Meadow, S. (*n* = 25). Information about Justice, L. M. (*n* = 64), Bishop, D. V. M. (*n* = 60), and Lawrence, L. (*n* = 59) has been introduced in the former section. Conti-Ramsden, G. did a lot of contribution to the development of young children with language disorders. Approximately, 77% of Conti-Ramsden, G.’s publications (*n* = 23 papers) were related to language disorder, and the factors affect language impairment. Goldin-Meadow, S. has put forward fundamental insights in many fields of cognitive science. There are numerous researchers that made great contributions to the CL field.

#### Organizations

**FIGURE 6:**
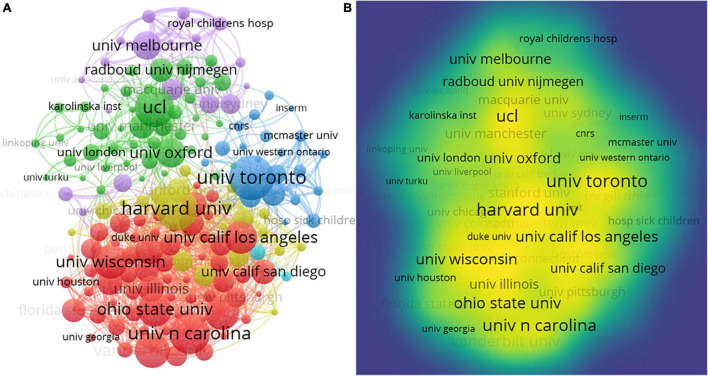
The visualization map of research organizations. **(A)** An organizational network visualization map; **(B)** a density visualization map.

VOSviewer was employed to give an organization citation visualization map to explore partnerships among the 16,003 organizations (Figure 6). For each of the 167 organizations, the total strength of the co-authorship links with other organizations was calculated. The organizations with the greatest total link strength were selected.

Figure 6A was conducted based on document weights. The 167 representative organizations were divided into 6 clusters and indicated by 6 colors with 5,632 links (Figure 6A). The total link strength is 22,321. Nodes or circle sizes represent the number of publications. The line between the two nodes demonstrated an academic link between the two organizations. The shorter the line, the stronger the link. The red cluster, at the left, gathered the largest number (*n* = 729) of organizations (Figure 6A). The red ball comes together to show that these institutions were working closely together. Of the organizations constituting this red cluster, the five organizations who had the most publications were: *University of Toronto* (*n* = 809), *Harvard University* (*n* = 767), *University of North Carolina* (*n* = 729), *University College London* (*n* = 713), and *Vanderbilt University* (*n* = 632). *Harvard University* has the strongest total link strength (*n* = 1,132) within this cluster. The *University of Toronto* has the second strongest total link strength (*n* = 1,006). These data show that these core institutions maintain close academic ties with other institutions.

Figure 6B was conducted based on total link weights. Organizations with a larger number of documents will have a larger and more obvious font size. The density visualization shows that those organizations, such as *University of Toronto, University of North Carolina, Ohio State University, University of California Los Angeles, University of Oxford*, and *University College London*, mainly lead CL research cooperation (Figure 6B). Ten organizations with the most publications were listed. When the threshold value was 110; there were 167 powerful organizations left in the CL research field (Supplementary Table 4).

The organization with the most publications is *University of Toronto* with the largest node, the most total documents (*n* = 890), and the second-largest total link strength (*n* = 928). The second most prolific organization was *Harvard University* (*n* = 767), which is cited most (*n* = 1,023). *University of North Carolina* has 729 TP and 33,466 total citations. Their cooperation was very close and has great influence in CL research.

#### Countries/Regions

**FIGURE 7:**
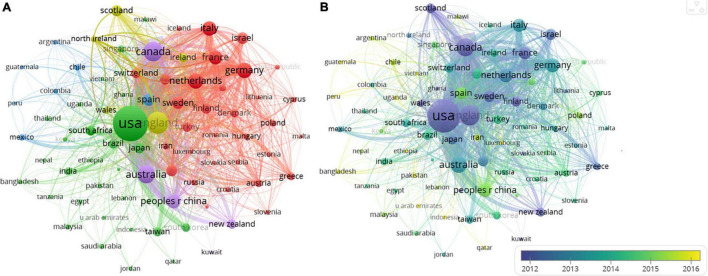
A cooperation network visualization map of countries/regions. **(A)** A network visualization map; **(B)** a density visualization map.

Countries and regions cooperation networks are visually mapped in Figure 7. The total strength of each country and its co-authorship links with other countries was calculated.

Link thickness represents collaboration strength, and the node size represents the number of publications from countries (Figure 7). The colors represent collaboration clusters. Five major clusters were identified. In Cluster 1 (red), Germany (*n* = 1,873), Netherlands (*n* = 1,845), Italy (*n* = 1,451), France (*n* = 1, 262), and Sweden (*n* = 993) co-authored a lot. In Cluster 2 (green), United States (*n* = 5,952), Japan (*n* = 416), Brazil (*n* = 456), and the Taiwan region of China (*n* = 248) were deeply linked to the cooperation in CL research. In Cluster 3 (blue), Spain (*n* = 1,087), Chile (*n* = 185), Mexico (*n* = 173), Argentina (*n* = 73), and Colombia (*n* = 70) has close cooperation with each other. In Cluster 4 (yellow), England (*n* = 5,336), Scotland (*n* = 631), Ireland (*n* = 326), and Wales (*n* = 255) kept a wide range of cooperation with other countries/regions. In Cluster 5 (purple), Canada (*n* = 3,659), Australia (*n* = 2,895), China (*n* = 1,539), and New Zealand (*n* = 452) cooperated closely with each other. Five clusters were closely related.

The main countries/regions co-operation characteristics are shown in Supplementary Table 5. United States ranks the first in terms of number of publications (*n* = 22,783) and the total link strength (*n* = 5,952). England is the second with 5,336 publications and 4,046 total links strength. It is worth mentioning that this paper only analyzes the papers written in English. Papers written in other languages, such as Spanish, French, and Chinese, deserve further study. The average publication year (APY) of the United States was 2010. While the APY of both China and Spain was 2015, indicating that Chinese and Spanish scholars have paid more and more attention to this topic.

### Co-word Analysis

Many research topics and themes have emerged and evolved in CL research over time. Data collected in the WoS database were evaluated to draw a network of co-occurring keywords and scientific categories in the CL research field. This section explores hot topics and keyword burst detection.

#### The Hot Topics: Keywords Co-occurrence Network

Keywords include nouns or phrases, which are efficient indexes to provide insight into research topics and research trends for the scientific field (Van Nunen et al., 2018). Keywords frequency analysis can be directly and effectively exploring the specific topics of the research field and core content. This section constructed some keyword co-occurrence networks and roughly outlined the CL research field.

**FIGURE 8:**
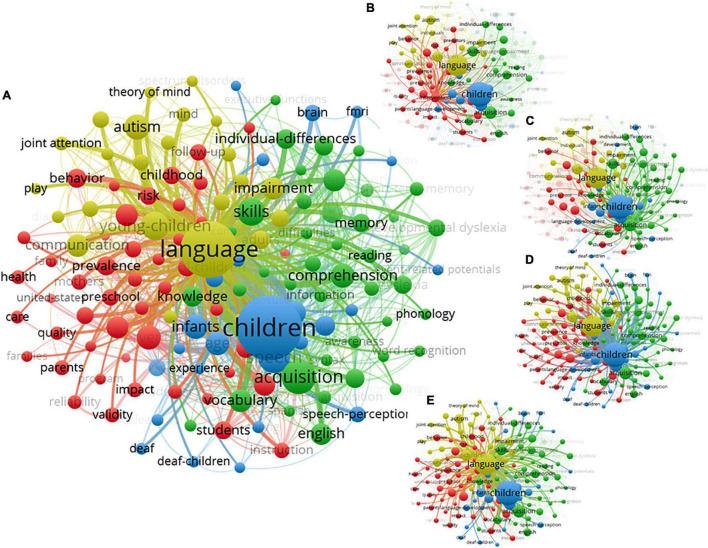
Co-keyword network visualizations. **(A)** Co-keyword network visualization of four clusters; **(B)** co-keyword network visualization of Cluster # 1; **(C)** co-keyword network visualization of Cluster # 2; **(D)** co-keyword network visualization of Cluster # 3; **(E)** co-keyword network visualization of Cluster # 4.

A co-occurrence network of all keywords was created by VOSviewer. For each keyword, the total strength of the co-occurrence links with other keywords was calculated. The strength of the link between two keywords represented the frequency of the two keywords being used simultaneously in the article. The keywords with the greatest total link strength were selected. Total link strength was 330,534. A total of 139 items were divided into four clusters with 9,205 links (Figure 8A).

In Figure 8A, node and label sizes represent the frequency of the keyword occurrences. The larger a circle, the greater number of times a keyword occurred. As expected, keywords “children,” “language,” “acquisition,” and “speech” occurred the most and had the strongest strength, and then followed by those high frequency keywords “young-children,” “skills,” “autism,” “infants,” “intervention,” “comprehension,” “age,” “communication,” “vocabulary,” etc. Different clusters are distinguished by colors. The distance between two keywords reflects the degree of interconnectedness and a topic similarity degree. The further the distance between the two keywords, the weaker their relation. The relatedness of keywords is calculated by counting the frequency of their occurrence together in the titles and abstracts in the same publications (Rodrigues et al., 2014). In Figure 8A, clusters are distinguished by different colors. Color indicates similar publication topics in the same cluster. Co-keyword network visualization is clearly displayed in four distinct clusters. By analyzing the four cluster major nodes, each cluster can be assigned an appropriate label.

Cluster # 1 (red) consists of 48 items with 138 links, and 9,811 total links strength (Figure 8B). The occurrence is 1,941. Cluster # 1 comprises keywords like “intervention,” “outcomes,” “adolescents,” “literacy,” “behavior,” “prevalence,” “disorders,” “students,” “preschool-children,” “risk,” “childhood,” “education,” “parents,” “kindergarten,” and “gender,” which focuses on factors influencing language acquisition.

Cluster # 2 (green) includes 58 items from 229 links, and 19,537 total link strength (Figure 8C). The occurrence is 3,756. Cluster # 2 comprises keywords like “acquisition,” “skills,” “comprehension,” “vocabulary,” “language impairment,” “working memory,” “English,” “knowledge,” “individual difference,” “phonological awareness,” “deficits,” “dyslexia,” “memory,” “developmental dyslexia,” “ability,” “reading,” “bilingualism,” “specific language impairment,” and “short-term memory,” which concentrates on language development in different aspects. CL is the key issue for linguists. The study of early childhood language acquisition has expanded from the first language (L1) to second-language (L2) acquisition. Whether L2 acquisition follows L1 acquisition rules, whether early childhood is the best age for L2 acquisition, and how L2 acquisition affects early childhood cognition, and how to carry out children’s L2 education reasonably and effectively are the research focus.

Cluster # 3 (blue) includes 56 items with 230 links, and 1,858 total link strength (Figure 8D). The occurrence is 2,233. Cluster # 3 comprises keywords like “children,” “speech,” “infants,” “age,” “performance,” “perception,” “language development,” “brain,” “recognition,” “patterns,” “development,” “discrimination,” “FMRI,” “cochlear implants,” “hearing,” “deaf,” “identification,” “experience,” and “identification,” which focuses on language diversity of children development. Researchers are more concerned about children’s performance in pronunciation, syntax, semantics, and vocabulary.

**FIGURE 9:**
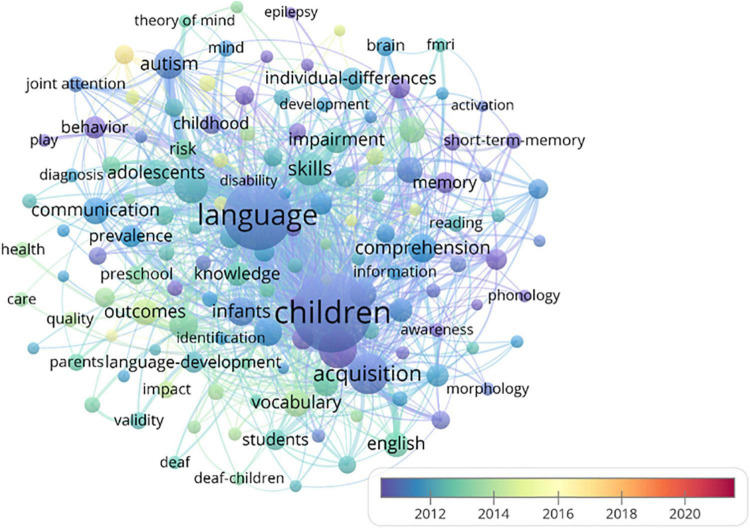
Co-keyword network visualization.

Cluster # 4 (yellow) consists of 40 items with 218 links, and 3,112 total link strength (Figure 8E). The occurrence is 558. Cluster # 4 comprises keywords like “young-children,” “autism,” “communication,” “impairment,” “adults,” “attention,” “preschoolers,” “autism spectrum disorder,” “individuals,” “toddlers,” “cognition,” “assessment,” “discourse,” “autism spectrum disorders,” and “theory of mind,” which focuses on different performance of both typically developing children and children with disabilities. The age range of the participants is relatively wide. For example, some participants are infants under 1 year of age (Oster and Werner, 2020), some are in the early stages of language development (Ylinen et al., 2016), some are over 10 years old (Nippold, 2006). Some children acquired a language in a purely native language setting; some children of immigrants have acquired a second language. Figure 9 shows the time-based overlay visualization of keyword co-occurrences based on the occurrences and average-publication-year score. The node color represents the average amount of keyword occurrences in a publication year.

The evolution of color from purple to red represents the variation of the hot topic over time. As shown in Figure 9, researchers focused on these keywords, such as “specific language impairment,” “intelligence,” “deficits,” “follow-up,” “disorders,” “phonology,” “discourse,” “readers,” “memory,” “disorder,” “disability,” “dyslexia,” etc. It showed that the terminology “specific language impairment” occurs mostly, since it has been used for many years, but, when not referring to it as a key word, it is expected to use “developmental language disorder” (Bishop et al., 2017). Since 2013, most articles have focused on “intervention,” “deaf children,” “risk,” “reliability,” and “literacy.” Since 2014, “autism spectrum disorder”, “school readiness”, and “oral language” had become the research hot topics, attracting the increasing attentions.

Supplementary Table 6 shows the information of the top 25 occurrence keywords. “Links” represents the co-occurrence of two keywords. The greater the total link strength, the stronger the link. Total link strength indicates the number of publications in which the two keywords appear simultaneously. New research hotspots mainly concentrated on “autism spectrum disorder,” “school readiness,” “oral language,” “reading comprehension,” “exposure,” “bilingualism,” “vocabulary,” “input,” “skills,” “kindergarten,” “cochlear implants,” and “intervention.” More and more research focuses on the individual difference in CL development and the importance of intervention in language education by typically developing children, and some are children with disabilities. Besides, child second language acquisition also attracts a lot of attention in the CL research field.

#### Burst Detection Analysis

**FIGURE 10:**
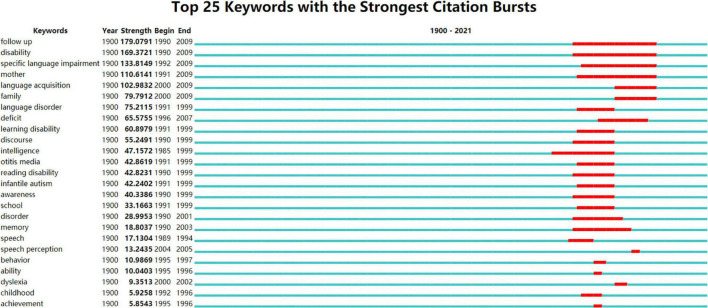
Twenty-five keywords with the strongest citation bursts.

Trending topics in CL research between 1900 and 2021 were analyzed by keyword burst analysis. Citation burst indicates that the scientific community during this period has or is paying special attention to certain specific issues over time. Keyword citation bursts refer to keywords that had a dramatic increase in the number of citations. Burst detection is an analysis method, searching keywords has attracted special attention of relevant scientific circles in a certain period. Keyword bursts indicate a sharp increase in the prominence of these keywords in the citation index and can partly reflect the dynamics of a scientific domain. Author and keyword bursts are important indicators for the researchers to identify the emerging or dying research trends (Kenekayoro, 2020). Burst detection is an effective analytical method to find the keywords of special concern to the relevant scientific community over a period. CiteSpace was used to conduct citation bursts analysis to detect intense CL research directions. Results showed the range was 1915–2021. Thirty-two keywords have citation bursts. Top 25 keywords with the strongest citation bursts between 1900 and 2021 were sorted by strength of bursts (Figure 10).

In Figure 10, “year” represents the starting time of the analysis; “strength” means the intensity of the bursts; “begin” means the starting year of the burst of keywords; “end” represents the end year of the burst of keywords. The red line represents the time period of significant change in the degree of burst. Fast-growing topics in the CL research field were detected. The keyword “follow up” (Freq.: 445, Burst: 179.08, Centrality: 0) has attracted the greatest attention from 1990 to 2009. This indicates that a lot of studies tend to explore the influences of early intervention experience in children on follow-up. Researchers tried to provide a prospective follow-up of typically developing children and children with disabilities.

The second strongest keyword is “disability” (Freq.: 421, Burst: 169.37, Centrality: 0), which has strongest burst during 1990–2009. Additionally, other keywords, including “specific language impairment,” (Freq.: 330, Burst: 133.81, Centrality: 0) “autism spectrum disorder,” (Freq.: 1,444, Burst: 110.04, Centrality: 0.29) “language disorder,” (Freq.: 121, Burst: 75.21, Centrality: 0) “learning disability,” (Freq.: 98, Burst: 60.9, Centrality: 0.07) “reading disability,” (Freq.: 69, Burst: 42.82, Centrality: 0), and “disorder” (Freq.: 1844, Burst: 29, Centrality: 0.04), have been trending topics since 1992. In addition to the research of typically developing children, the language acquisition of children with disabilities also attracts much attention. Since 1996, the language development of children with disabilities has become the main field of CL research, particularly the study of “deficit,” “otitis media,” “infantile autism,” and “dyslexia.” The theme of the research changes quickly with time. Factors that would affect the CL acquisition are getting more and more attention, such as “mother,” “family,” “school,” “childhood,” “toddler,” and “brain.” Performance of CL also attracts considerable attention, such as “discourse,” “awareness,” “memory,” “speech,” “speech perception,” “behavior,” “ability,” and “achievement.” Specific research on “awareness” was a hot topic, such as “phonological awareness,” “morphological awareness,” and “phonemic awareness.” The keyword with the earliest burst was “intelligence,” which occurred in 1985.

**FIGURE 11:**
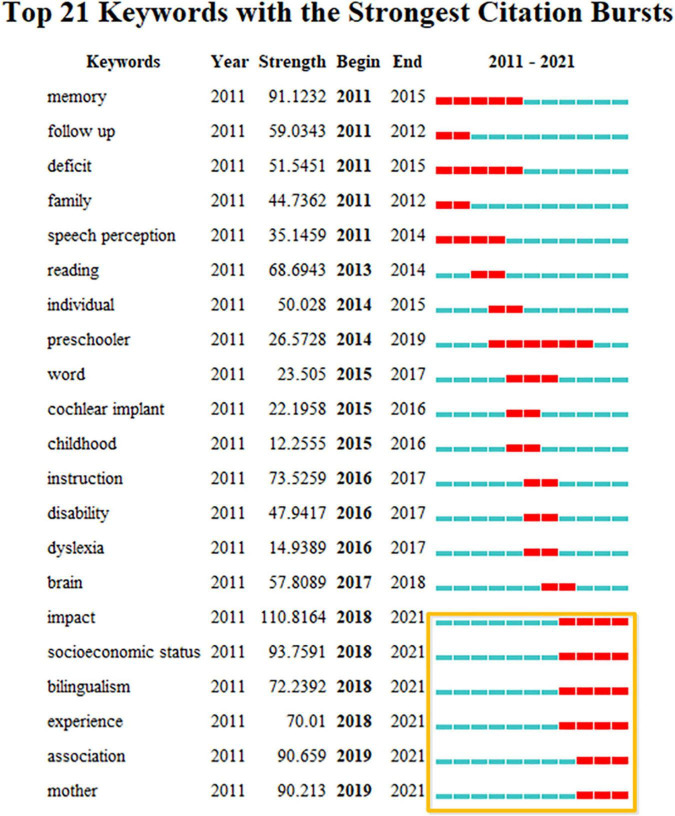
Twenty-one keywords with the strongest citation bursts between 2000 and 2020 about CL research sorted by strength.

What have been the hot keywords in recent 10 years? The author also conducted a keyword burst detection analysis about the last decade. Twenty-one most common keywords having the strongest citation bursts during the last 10 years have been sorted by strength, starting time, and duration (Figure 11). The red line represents the citation burst period, indicating the fastest growing topics in this field.

The keywords framed in orange have been hot topics since 2018 (Figure 11). The topics, such as “memory,” “follow-up,” “deficit,” “family,” “speech perception,” “reading,” “individual,” “preschooler,” “word,” “cochlear implant,” “childhood,” “instruction,” and “disability” attracted great attention. Since 2018, “impact,” “socioeconomic status,” “bilingualism,” “experience,” “association,” and “mother” have become new focuses of research. Among these terms, the keyword “preschooler” lasted the longest and received close attention of researchers, 5 years to be exact.

### Co-citation Analysis

Each paper usually quotes many references, which are represented as nodes in the co-citation visualization network. The links between nodes indicate how often they reference in the same article. The hypothesis is that if two references are often co-cited, it may indicate that the two references are related in some aspects. The double relationship between cited references and their citing papers can be found through the connection between literature. Chen et al. (2012) proposed that the co-citation network formed in this way can identify the research focus of the potential scientific community.

Co-citation refers to the number of instances of two items (such as authors, documents, or journals) cited in the third article (Chen, 2006). This paper analyzes the index bibliographic records in WoS and establishes the journal co-citation network, author co-citation network, and reference co-citation network. This section analyzes co-citation analyses of journals, authors, and articles.

#### Journal Co-citation Network

**FIGURE 12:**
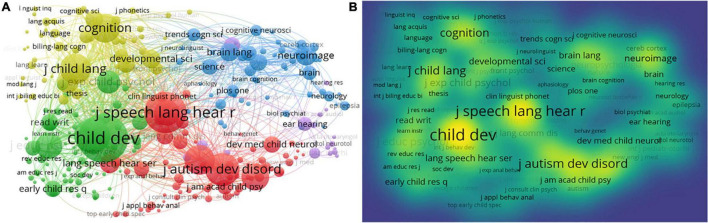
Journal co-citation networks of CL. **(A)** Journal co-citation network visualization; **(B)** journal co-citation density visualization.

Journal co-citation network was conducted using VOSviewer. The network visualization appears in Figure 12.

Journals with higher co-citation frequency have larger fonts, which are more prominent in Figure 12. They are *Child Development* (citation number = 51,394), *Journal of Speech Language and Hearing Research* (*n* = 40,071), *Developmental Psychology* (*n* = 32,092), *Journal of Autism and Developmental Disorders* (*n* = 29,678), and *Journal of Child Language* (*n* = 25,778). These journals have contributed significantly to CL research. The citations of these journals are more than the others in the CL field. The top 10 journals in the co-citation network are shown in Supplementary Table 7. The link strength of the top 10 journals is large. There are more than 19,000 citations of the top 10 journals, respectively. These journals are very prolific and have made great contributions to the CL research.

#### Author Co-citation Analysis

**FIGURE 13:**
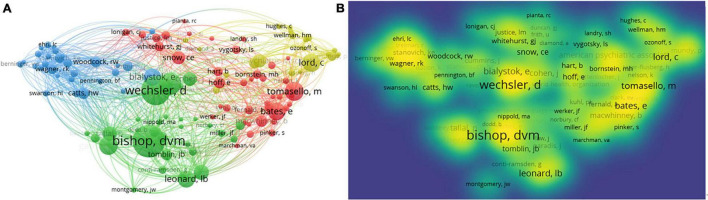
Author co-citation networks of CL. **(A)** Author co-citation network visualization; **(B)** author co-citation density visualization.

Author co-citation network was performed by VOSviewer. The network visualization is shown in Figure 13.

The 120 items were divided into 4 clusters with 6,969 links (Figure 13). The total link strength was 974,646. The co-citation frequency of the top five most co-cited authors appears in Figure 13. They are “Bishop, D. V.,” “Wechsler, D.,” “Dunn, L. M.,” “Tomasello, M.,” and “Gathercole S. E.” Supplementary Table 8 shows the top 10 authors in the co-citation network. All the top 10 authors have been cited more than 3,000 times. These authors have contributed a lot to the research and development of CL.

#### Reference Co-citation Analysis

Reference co-citation analysis evaluates references cited by references in the CL field. Co-citation refers to that when two references are cited in an article. Reference co-citation analysis was conducted by VOSviewer. For each of the 175 cited references, the total strength of the co-citation links with other cited references will be calculated. The cited references with the greatest total link strength will be selected.

**FIGURE 14:**
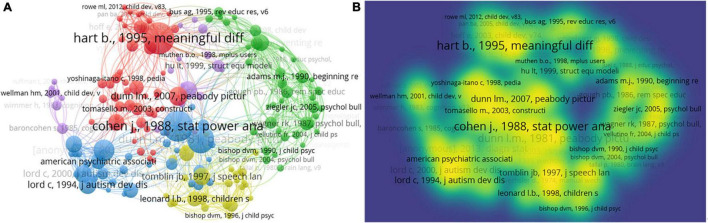
Reference co-citation networks of CL were based on citation weights. **(A)** Reference co-citation network visualization; **(B)** reference co-citation density visualization.

The 869,771 bibliographic recordings from 1900 to 2021 are visualized in Figure 14. One hundred and seventy-five items were divided into 5 clusters with 11,915 links. The total link strength is 126,566. The nodes and links represent the cited references and co-citation relationships among the bibliometric data. Supplementary Table 9 presents the top 10 most cited references in the CL field.

The work that has been cited most was Hart and Risley (1995). Hart and Risley (1995) conducted a longitudinal study to record and analyze verbal interactions of 42 children with their families from the time they first began to say words (about 1 year) until 3 years old. They explored language development in young children and how home experience influences child development. Results showed that the economic condition of children’s homes and the amount of language experience were the main factors that affect CL acquisition. The second work cited most was Cohen (1988). Cohen (1988) published a book named “*Statistical Power Analysis for the Behavioral Sciences*.” Researchers cite this reference as the formula they use to compute effect size. This suggests that empirical research accounts for the majority in the field of children’s language acquisition. The third work cited most was Dunn and Dunn (1981), named “*The Peabody Picture Vocabulary Test*.” Dunn, L. M. published the first Peabody Picture Vocabulary Test (PPVT) scale in 1959. Then, Dr. Dunn, L. M. and his wife, Dunn, L., published several revisions to the PPVT test. His son, Dunn, D. M., is the co-author of the PPVT-4. The PPVT-4 is widely used for assessing vocabulary acquisition. The test is used to measure the understanding of vocabulary of children and adults. In sum, all these classic pieces of literature provide a lot of ideas and inspiration for the academic community to explore how children acquire language.

## Discussion

Using the bibliometric analysis method, this study has analyzed the publications in the past 121 years. The research shows a multi-disciplinary integration trend. Researchers have systematically analyzed and explained the process and essence of children’s language acquisition from the perspectives of linguistic, psychology experimental, psychology developmental, rehabilitation, language linguistics, and other disciplines. The theory and experimental methods of language acquisition are constantly updated, which makes the research in this field flourish. In recent years, the maturity of language acquisition theories and methods has led to a sharp increase in the number of publications and citations on related topics. The research methods also show a trend from putting forward a single theoretical hypothesis to the combination of theory and experiment. Many researchers used the observation method, corpus-based approach, case study, longitudinal study, and horizontal study to explore various elements of children’s language development, including the phonological, lexical, and syntactic analysis. Especially, the cognitive perspective of the interaction between external environmental stimuli and children’s language processing initiative has attracted extensive attention.

The aim and the scope of most mainstream journals were related to the children with language disorders. The mainstream categories were linguistic and psychology. The nodes of diverse disciplines are closely connected, forming a clear, multi-directional, and multi-disciplinary citation spatial network structure. Most publications in linguistics focus on language acquisition, second language acquisition, language development, pronunciation, vocabulary, grammar, syntax, pragmatics, semantics, and language ability. It is the core discipline of children’s language research. The second is psychology, mainly related to developmental psychology and experimental psychology. It mainly studies children’s speech behavior observation, language ability test, working memory, knowledge, learning, and other fields. In terms of rehabilitation, research focused on special children or vulnerable children, carried out research on the diagnosis and treatment of children with language disorder and hearing impairment, as well as the screening, diagnosis, and rehabilitation of pathological language loss of children with high and low functional autism, to verify the groups whose language development is lower than the norm of typically developing children. Many researchers focus on pedagogy, such as educational environment creation, educational intervention, cultural resources, early reading, personal interaction, peer communication, follow-up, and so on.

Cross-disciplinary collaboration requires the researchers should not only master linguistic theories and experimental methods of traditional language research methods but also be good at cooperating with researchers of other relevant disciplines to understand the broader relationship between language, mind, and brain in a cross-disciplinary way. The cooperation and exchange between scholars from different research fields can stimulate new ideas and make more innovative discoveries. Opportunities and challenges coexist. Differences in academic backgrounds may create obstacles in the dialogue between scholars. For example, there were still great disputes and differences in the research on the internal mechanism of language acquisition and how the external environmental factors work. To explore the main issues and focus of debate in the CL research field, researchers should not only master linguistic theories and experimental methods of cognitive science but also be good at cooperating with researchers in other disciplines, such as cognitive science, developmental psychology, neuroscience, artificial intelligence, etc.

The main researchers, institutions, countries, and journals with the largest number of papers or cited frequency come from developed countries. They paid more attention to use some new experimental methods to explore how children acquire language and the influencing factors. The most concerned problem of authors with large number of articles or high citation rate is to study the language development of children with language disabilities. Besides, empirical papers were found to be cited more often. It showed to be noted that citation frequency can reflect popularity of a journal or the head of a lab more than quality of research (Aksnes et al., 2019). In sum, the modern study of the CL benefits greatly from the progress of methodology and conceptualism proposed from cooperation. It is worth mentioning that all the authors have made significant contributions to CL research.

Research trend analysis can provide some insights for researchers and educational practitioners to identify which are important research directions. Through content analysis, it is found that researchers pay attention to these topics: (1) children’s language ability testing and development, language behavior observation, and strategies; (2) the internal mechanism and physiological characteristics of children’s language development; (3) language diversity of children development; (4) performance of typically developing children and children with developmental language disorder, such as cognition and medical clinical diagnosis; (5) empirical research based on data analysis; (6) children’s language policy and language teacher education; and (7) the language phenomenon of bilingual children has attracted much attention of academic circles. Burst detection analysis showed that the prominent topics of high concern in the latest academic circles (2018–2021) were as follows: “impact,” “socioeconomic status,” “bilingualism,” “experience,” “association,” and “mother.” These keywords focus on the influence factors of language acquisition. Language development is limited by many environmental factors. For instance, in addition to the influence of parents, the social environment is also influenced by children’s peers and other adults. Physical environment may play a significant role in the development of CL. Continuous language assessment is critical to ensure timely intervention to prevent language disorders in children. More research is needed on specific environmental factors and their relationship with children’s language development.

Foreign research focuses on the internal and external mechanisms of children’s individual language from macro policy-making to micro. The research scope is broad and involves rich research contents. The research methods include qualitative, quantitative, and mixed research. Topics related to children’s language education and language diagnosis and rehabilitation of children with language disorders were the research focus. More and more researchers pay attention to the language development and education of disadvantaged children. In short, the results of bibliometric analysis show that the research field of children’s language development is extensive, cross-disciplinary.

This study will help readers understand the dynamic development trend of CL from research results. It will help scholars quickly identify the hot spots and focus issues of CL research, guide them to find the most influential references, and choose the most influential or relevant researchers and institutions to cooperate. Through the analysis of the results, it will help researchers to find the main journal contribution direction and promote the further development of research achievements in scientific research institutions.

## Conclusion

Under the impact of new empirical materials and new theories, the field of CL is undergoing profound changes. This paper makes a bibliometric analysis of the research trend and evolution of child language research in the past 100 years. Results showed that, especially, in the past two decades, the attention of child language research has been very high, and the relative publication amount has increased sharply. This study visualized the most productive and leading journals and disciplines. Cross-disciplinary collaboration can not only solve the shackles caused by the complexity of children’s language acquisition research but also mobilize the knowledge reserve of each involved field and give full play to their expertise. The collaboration between researchers and educational practitioners would grow.

The co-authorship analysis shows that multi-author publications account for a large proportion. Research of is topic involves a wide range of disciplines. This shows the broad theme and multidisciplinary nature of CL research. In addition, the most prolific organizations and countries were displayed. Keyword co-occurrence showed that the theme of the CL study changes quickly. This shows that researchers pay more and more attention to compare the typical development of children and children with language disorders. Co-citation analysis shows the leading co-cited journals, the major co-cited authors, and the most influential co-cited references. The most cited papers are those about experimental methods and theories, laying the foundation for the follow-up research.

Findings of this study will contribute to the research in this field. However, there are some limitations of this study. Firstly, the datum collection of this research was limited to the research paper indexed by SSCI, SCIE, and A&HCI, written in English in WoS. Other databases, such as PubMed or Scopus, deserve attention. Only journal articles in CL were used for the bibliometric analysis. Other types such as dissertations, books, and conference papers may be investigated in the future study. Bibliometric analysis on previous work provided some suggestions and valuable references for researchers in the CL field.

## Data Availability Statement

The original contributions presented in the study are included in the article/Supplementary Material; further inquiries can be directed to the corresponding author.

## Author Contributions

XG formulated the research questions, collected the data, conducted the bibliometric analysis, wrote the draft of the manuscript and revised it. The author approved the submitted version.

## Conflict of Interest

The author declares that the research was conducted in the absence of any commercial or financial relationships that could be construed as a potential conflict of interest.

## Publisher’s Note

All claims expressed in this article are solely those of the authors and do not necessarily represent those of their affiliated organizations, or those of the publisher, the editors and the reviewers. Any product that may be evaluated in this article, or claim that may be made by its manufacturer, is not guaranteed or endorsed by the publisher.
