# Supplementary material for: A Bibliometric Analysis of Child Language During 1900–2021
Source: Front Psychol. 2022 Jun 8;13:862042. doi: 10.3389/fpsyg.2022.862042 (PMC9216231; doi:10.3389/fpsyg.2022.862042)
Supplement: Supplementary file 1 [file Table_1.docx]

Supplementary Material

# Supplementary Tables

# Supplementary Table 1. Categories chosen for analysis in WoS.

| Rank | Category | Rank | Category | Rank | Category |
| --- | --- | --- | --- | --- | --- |
| 1 | Linguistics | 20 | Multidisciplinary Sciences | 39 | Psychology Biological |
| 2 | Rehabilitation | 21 | Genetics Heredity | 40 | Acoustics |
| 3 | Psychology Developmental | 22 | Surgery | 41 | Computer Science Interdisciplinary Applications |
| 4 | Audiology Speech Language Pathology | 23 | Medicine General Internal | 42 | Literature |
| 5 | Psychology Experimental | 24 | Family Studies | 43 | Physiology |
| 6 | Education Educational Research | 25 | Social Work | 44 | Computer Science Information Systems |
| 7 | Pediatrics | 26 | Neuroimaging | 45 | Respiratory System |
| 8 | Education Special | 27 | Health Care Sciences Services | 46 | Biology |
| 9 | Language Linguistics | 28 | Psychology Social | 47 | Music |
| 10 | Psychology Educational | 29 | Communication | 48 | Psychology Applied |
| 11 | Neurosciences | 30 | Radiology Nuclear Medicine Medical Imaging | 49 | Computer Science Theory Methods |
| 12 | Clinical Neurology | 31 | Social Science Interdisciplinary | 50 | Education Scientific Disciplines |
| 13 | Psychiatry | 32 | Sociology | 51 | Humanities Multidisciplinary |
| 14 | Otorhinolaryngology | 33 | Medicine Research Experimental | 52 | Critical Care Medicine |
| 15 | Psychology Multidisciplinary | 34 | Nursing | 53 | Developmental Biology |
| 16 | Psychology | 35 | Dentistry Oral Surgery Medicine | 54 | Psychology Psychoanalysis |
| 17 | Public Environmental Occupational Health | 36 | Anthropology | 55 | Computer Science Software Engineering |
| 18 | Behavioral Sciences | 37 | Computer Science Artificial Intelligence | 56 | Social Issues |
| 19 | Psychology Clinical | 38 | Social Sciences Biomedical | 57 | Primary Health Care |

# Supplementary Table 2. Top 10 cited source journals about CL research.

| Rank | Journal | Links | TS | TP | Citations | IF | JCR category |
| --- | --- | --- | --- | --- | --- | --- | --- |
| 1 | Journal of Speech Language and Hearing Research | 219 | 41379 | 1381 | 51331 | 1.873 | Rehabilitation \| Audiology & Speech-Language Pathology \| Rehabilitation \| Linguistics; Rehabilitation\| Social Sciences, General |
| 2 | Child Development | 217 | 16932 | 499 | 40774 | 4.891 | Psychology \| Educational \| Psychology \| Developmental |
| 3 | Journal of Autism and Developmental Disorders | 201 | 13542 | 830 | 40738 | 1.43 | Psychology \| Developmental |
| 4 | Pediatrics | 191 | 4796 | 421 | 30467 | 2.77 | Pediatrics \| Medical Research, General Topics \| Clinical Medicine |
| 5 | Developmental Psychology | 210 | 12015 | 421 | 26632 | 3.063 | Psychology\| Developmental, Psychiatry |
| 6 | Journal of Child Language | 203 | 15484 | 796 | 21968 | 1.62 | Psychology\| Developmental \| Psychology, Experimental \| Linguistics\| Psychiatry |
| 7 | Journal of Child Psychology and Psychiatry | 209 | 8225 | 264 | 21022 | 7.035 | sychiatry \| Psychology \| Psychology, Developmental\| Clinical Psychology & Psychiatry |
| 8 | Cognition | 182 | 7062 | 331 | 19510 | 3.294 | Psychology, Experimental\| Psychiatry |
| 9 | Journal of Educational Psychology | 182 | 7062 | 331 | 19510 | 5.028 | Psychology \| Educational; Psychiatry |
| 10 | Applied Psycholinguistics | 212 | 12740 | 494 | 16338 | 1.412 | Psychology, Experimental \| Linguistics\| Psychiatry/ |

Note: TS= Total link strength; TP= Total publications; IF= 2019 Impact Factor; Data Source: WoS.

# Supplementary Table 3. The top 10 strong co-authorship linked document-productive authors

| Rank | Label | Cluster | Links | TS | TD | TC | NC | APY | AC | AN | A1 |
| --- | --- | --- | --- | --- | --- | --- | --- | --- | --- | --- | --- |
| 1 | Justice, L.M. | 7 | 15 | 205 | 172 | 4838 | 240.35 | 2014 | 28.13 | 1.40 | 64 |
| 2 | Bishop, D.V.M. | 8 | 6 | 24 | 69 | 2968 | 96.78 | 2011 | 43.01 | 1.40 | 60 |
| 3 | Leonard, L.B. | 3 | 6 | 36 | 69 | 1671 | 64.85 | 2013 | 24.22 | 0.94 | 59 |
| 4 | Verhoeven, L. | 21 | 3 | 65 | 126 | 1726 | 96.47 | 2014 | 13.70 | 0.77 | 11 |
| 5 | Conti-Ramsden, G. | 13 | 9 | 85 | 67 | 2167 | 88.21 | 2013 | 32.34 | 1.32 | 30 |
| 6 | Tomasello, M. | 15 | 6 | 43 | 64 | 3000 | 93.45 | 2014(2012) | 37.58 | 1.46 | 16 |
| 7 | Reilly, S. | 4 | 14 | 120 | 81 | 1623 | 99.26 | 2016 | 20.04 | 1.23 | 8 |
| 8 | Tager-Flusberg, H. | 1 | 11 | 41 | 64 | 2633 | 139.47 | 2014 | 41.14 | 2.18 | 13 |
| 9 | Snowling, M. J. | 9 | 3 | 45 | 62 | 2233 | 137.14 | 2014 | 36.02 | 2.21 | 15 |
| 10 | Goldin-Meadow, S. | 5 | 4 | 25 | 74 | 2860 | 126.23 | 2013.78 | 138.65 | 1.71 | 25 |

Note: TS: total link strength; TD: total documents; TC: total citations; NC: norm. citations; APY: avg. pub. Year; AC: avg. citations; AN：avg. norm. citations. A1：The first author

# Supplementary Table 4. The top 10 strong co-authorship linked document-productive organizations

| Label | Cluster | | Links | TS | TD | TC | NC | APY | AC | AN |
| --- | --- | --- | --- | --- | --- | --- | --- | --- | --- | --- |
| University of Toronto | 3 | | 128 | 928 | 809 | 26,544 | 1,045.94 | 2012 | 32.81 | 1.29 |
| Harvard University | 4 | | 142 | 1,023 | 767 | 36,282 | 1,368.13 | 2011 | 47.30 | 1.78 |
| University of North Carolina | | 1 | 102 | 748 | 729 | 33,466 | 1,075.34 | 2011 | 45.91 | 1.48 |
| University College London | | 2 | 111 | 615 | 713 | 25,797 | 1,055.35 | 2013 | 36.18 | 1.48 |
| Vanderbilt University | | 1 | 103 | 632 | 632 | 21,446 | 1,006.83 | 2012 | 33.93 | 1.59 |
| Ohio State University | | 1 | 103 | 545 | 620 | 12,587 | 649.63 | 2014 | 20.30 | 1.05 |
| University of Washington | | 1 | 118 | 735 | 616 | 24,501 | 940.13 | 2010 | 39.77 | 1.53 |
| University of Wisconsin | | 1 | 104 | 531 | 614 | 21,256 | 706.41 | 2011 | 34.62 | 1.15 |
| University of California | | 1 | 120 | 624 | 587 | 27,000 | 963.11 | 2010 | 46.00 | 1.64 |
| University of Oxford | | 2 | 103 | 502 | 531 | 24,044 | 864.74 | 2011 | 45.28 | 1.63 |

Note: TS: total link strength; TD: total documents; TC: total citations; NC: norm. citations; APY: Avg. pub. year; AC: avg. citations; AN: Avg. norm. citations.

# Supplementary Table 5. The topmost 10 strong co-authorship linked document-productive countries/regions

| RO^a^ | Countries/regions | Clusters | Links | Total link strength | Documents | Citations | APY^b^ |
| --- | --- | --- | --- | --- | --- | --- | --- |
| 1 | USA | 2 | 77 | 5952 | 22783 | 697456 | 2010 |
| 2 | England | 4 | 77 | 4046 | 5336 | 184135 | 2011 |
| 3 | Canada | 5 | 72 | 2238 | 3659 | 109686 | 2011 |
| 4 | Australia | 5 | 70 | 1951 | 2895 | 60624 | 2013 |
| 5 | Germany | 1 | 68 | 1880 | 1873 | 43756 | 2013 |
| 6 | Netherlands | 1 | 67 | 1643 | 1845 | 39247 | 2013 |
| 7 | Peoples R China | 5 | 66 | 1178 | 1539 | 23610 | 2015 |
| 8 | Italy | 1 | 62 | 1169 | 1451 | 31107 | 2013 |
| 9 | France | 1 | 59 | 1289 | 1262 | 33873 | 2012 |
| 10 | Spain | 3 | 63 | 1008 | 1087 | 16107 | 2015 |

Note: ^a^ RO: Ranking Order; ^b^ APY: Average Publication Year.

# Supplementary Table 6. The link and total link strength of the top 25 occurrence key words

| Rank | Key words | Cluster | Links | TS^b^ | Occurrences | APY ^c^ |
| --- | --- | --- | --- | --- | --- | --- |
| 1 | children | 3 | 138 | 50447 | 15263 | 2011 |
| 2 | language | 4 | 138 | 43430 | 11896 | 2012 |
| 3 | acquisition | 2 | 138 | 17000 | 4319 | 2012 |
| 4 | speech | 3 | 138 | 15656 | 3901 | 2011 |
| 5 | young-children | 4 | 138 | 12806 | 3105 | 2012 |
| 6 | skills | 2 | 138 | 15024 | 3012 | 2013 |
| 7 | autism | 4 | 138 | 9883 | 2443 | 2012 |
| 8 | infants | 3 | 137 | 8662 | 2281 | 2012 |
| 9 | intervention | 1 | 138 | 10203 | 2239 | 2013 |
| 10 | comprehension | 2 | 138 | 10205 | 2224 | 2012 |
| 11 | age | 3 | 138 | 9088 | 2207 | 2012 |
| 12 | communication | 4 | 137 | 8673 | 2014 | 2012 |
| 13 | vocabulary | 2 | 138 | 10228 | 1999 | 2014 |
| 14 | performance | 3 | 138 | 8320 | 1971 | 2012 |
| 15 | language impairment | 2 | 138 | 8153 | 1913 | 2012 |
| 16 | outcomes | 1 | 138 | 8049 | 1905 | 2014 |
| 17 | impairment | 4 | 138 | 8355 | 1900 | 2013 |
| 18 | adolescents | 1 | 138 | 6912 | 1880 | 2013 |
| 19 | working-memory | 2 | 135 | 8723 | 1867 | 2014 |
| 20 | English | 2 | 137 | 6919 | 1831 | 2013 |
| 21 | literacy | 1 | 137 | 8043 | 1706 | 2013 |
| 22 | perception | 3 | 138 | 6297 | 1586 | 2012 |
| 23 | knowledge | 2 | 138 | 6640 | 1555 | 2013 |
| 24 | Individual-differences | 2 | 138 | 6771 | 1487 | 2012 |
| 25 | behavior | 1 | 137 | 5383 | 1434 | 2011 |

Note: ^a^ RO: Ranking Order; ^b^ TS: Total link strength; ^c^APY: Average Publication Year.

# Supplementary Table 7. The top 10 journals in the co-citation network.

| Label | Cluster | Links | Total Link Strength | Citations |
| --- | --- | --- | --- | --- |
| Child Development | 2 | 280 | 1728349 | 51394 |
| Journal of Speech Language and Hearing Research | 1 | 280 | 1405268 | 40071 |
| Developmental Psychology | 2 | 280 | 1189953 | 32092 |
| Journal of Autism and Developmental Disorders | 1 | 280 | 934277 | 29678 |
| Journal of Child Language | 4 | 280 | 816668 | 25778 |
| Journal of Child Psychology and Psychiatry | 1 | 280 | 902666 | 25081 |
| Cognition | 4 | 280 | 947295 | 24186 |
| Pediatrics | 1 | 280 | 466098 | 21884 |
| Journal of Educational Psychology | 2 | 280 | 770591 | 21056 |
| Journal of Speech and Hearing Research | 1 | 280 | 604690 | 19884 |

# Supplementary Table 8. The top 10 authors in the co-citation network.

| Label | Cluster | Links | Total Link Strength | Citations |
| --- | --- | --- | --- | --- |
| Bishop, D. V. M | 2 | 119 | 76,546 | 7,367 |
| Wechsler, D. | 2 | 119 | 47,311 | 6,586 |
| Dunn, L. M. | 2 | 119 | 41,185 | 5,068 |
| Tomasello, M. | 1 | 118 | 32,953 | 4,249 |
| Gathercole, S. E. | 2 | 119 | 47,148 | 4,205 |
| Lord, C. | 4 | 117 | 31,382 | 4,088 |
| Bates, E. | 1 | 119 | 34,900 | 3,947 |
| Bialystok, E. | 3 | 117 | 22,985 | 3,889 |
| Leonard, L. B. | 2 | 119 | 40,287 | 3,879 |
| Rice, M. L. | 2 | 119 | 39,041 | 3,447 |

# Supplementary Table 9. The top 10 most cited references in the co-citation network.

| **Label** | **Title** | **Cluster** | **Links** | **Total link strength** | **Citations** |
| --- | --- | --- | --- | --- | --- |
| Hart & Risley (1995) | Meaningful Differences in Everyday Experience of Young American Children | 1 | 172 | 6391 | 1521 |
| Cohen (1988)^a^ | Statistical power analysis for the behavioral sciences | 3 | 174 | 4140 | 1486 |
| Dunn and Dunn (1981) | The Peabody Picture Vocabulary Test | 3 | 173 | 3697 | 1330 |
| American Psychological Association (2013) | Diagnostic and statistical manual of mental disorders: DSM-5™ (5th ed.) | 3 | 158 | 2456 | 1025 |
| Lord et al. (1994) | Autism Diagnostic Interview-Revised: A revised version of a diagnostic interview for caregivers of individuals with possible pervasive developmental disorders | 1 | 167 | 2838 | 908 |
| Tomblin et al. (1997) | Prevalence of specific language impairment in kindergarten children. | 3 | 138 | 2803 | 870 |
| Mullen E.M. (1995) | Mullen Scales of Early Learning | 5 | 167 | 3108 | 837 |
| Snow et al. (1998) | Preventing Reading Difficulties in Young Children | 3 | 141 | 2717 | 836 |
| Fenson et al. (1993) | MacArthur Communicative Development Inventories | 4 | 171 | 3182 | 823 |

Note: Cohen (1988)^a^ reference is a book on (statistical) effect size, not child language. Rather, researchers cite this reference as the formula they use to compute effect size.

Dunn^b^ and his wife and son have published several revisions of ^“^The Peabody Picture Vocabulary Test” (PPVT).

# Reference:

American Psychiatric Association. (2013). Diagnostic and statistical manual of mental disorders: DSM-5 (5th ed.). Washington, DC: American psychiatric association.

Cohen, J. (1988). Statistical Power Analysis for the Behavioural Science (2nd Edition). In *Statistical Power Anaylsis for the Behavioral Sciences*.

Dunn, L. M., and Dunn, L. M. (1981). *Peabody Picture Vocabulary Test-Revised.* Circles Pines, MN: American Guidance Service.

Fenson, L., Dale, P. S., Reznick, J. S., Thal, D., Bates, E., Hartung, M. S., Pethick, S., & Reilly, J. S. (1993). *MacArthur Communicative Development Inventories*.

Hart, B., & Risley, T. R. (1995). Meaningful Differences in Everyday Experience of Young American Children. *Brookes Publishing Company, Inc., P.O. Box 10614, Baltimore, MD 21285-0624.*

Lord, C., Rutter, M., & Couteur, A. L. (1994). Autism Diagnostic Interview-Revised: A revised version of a diagnostic interview for caregivers of individuals with possible pervasive developmental disorders. *Journal of Autism & Developmental Disorders*, *24*(5), 659–685.

Mullen, E. M. (1995). Mullen Scales of Early Learning. Circle Pines, MN: American Guidance Service.

Snow, C. E., Burns, M. S., & Griffin, P. (1998). Preventing Reading Difficulties in Young Children. *Beginning Reading*.

Tomblin, J., Bruce, Records, Nancy, L., Buckwalter, Paula, Xuyang, & Zhang. (1997). Prevalence of specific language impairment in kindergarten children. *Journal of Speech, Language & Hearing Research*, *40*(6), 1245.
